# Chromatin dynamics at the maternal to zygotic transition: recent advances from the zebrafish model

**DOI:** 10.12688/f1000research.21809.1

**Published:** 2020-04-28

**Authors:** Bagdeser Akdogan-Ozdilek, Katherine L Duval, Mary G Goll

**Affiliations:** 1 Department of Genetics, University of Georgia, Athens, GA, USA

**Keywords:** chromatin, transcription, maternal-to-zygotic transition, zebrafish, histones, DNA methylation

## Abstract

Early animal development is characterized by intense reorganization of the embryonic genome, including large-scale changes in chromatin structure and in the DNA and histone modifications that help shape this structure. Particularly profound shifts in the chromatin landscape are associated with the maternal-to-zygotic transition, when the zygotic genome is first transcribed and maternally loaded transcripts are degraded. The accessibility of the early zebrafish embryo facilitates the interrogation of chromatin during this critical window of development, making it an important model for early chromatin regulation. Here, we review our current understanding of chromatin dynamics during early zebrafish development, highlighting new advances as well as similarities and differences between early chromatin regulation in zebrafish and other species.

## Overview

Animal genomes undergo a period of intense reorganization during early development as zygotic transcription initiates and embryos transition from a totipotent to a lineage-committed state. This reorganization is reflected by dramatic changes in chromatin structure and in the DNA and histone modifications that help drive this structure. During the last two decades, zebrafish have become an important and established model for studying chromatin in the context of vertebrate development. Two recent comprehensive reviews detail the many seminal insights into chromatin regulation that have been obtained using zebrafish ^1, 2^. This focused review highlights current advances in our understanding of chromatin dynamics during early zebrafish embryogenesis. We provide an emerging picture of chromatin changes during the awakening of the zebrafish zygotic genome, integrate this knowledge with new data gained from other species, and highlight research directions where the strengths of the zebrafish model provide high potential for new advances.

### Zebrafish

Zebrafish offer a powerful system for studying chromatin transitions associated with early development. External fertilization means that embryos are accessible for observation, manipulation, and molecular interrogation from the 1-cell stage onward without surgical intervention. Following fertilization, zebrafish embryos develop quickly over the next 24 hours, moving through a stereotypical program defined by cleavage, blastula, gastrula, and segmentation periods. During this time, embryos can be accurately staged on the basis of morphological characteristics as well as time post fertilization ^3^. Early cleavage stages exhibit several unique features, including a high ratio of cytoplasmic to nuclear volume, rapid cell divisions in which the entire genome is replicated in less than 15 minutes, and sac-like vesicles called karyomeres that transiently encase individual or groups of chromosomes near the end of mitosis ^4– 7^. Zebrafish display a conserved fate map following blastula stages and undergo further cell fate restriction during gastrulation ^8^.

## Awakening of the genome

The maternal-to-zygotic transition (MZT) represents a critical milestone in early animal embryogenesis. During this transition, developmental control shifts from maternally provided proteins and RNAs to zygotic transcripts ^9– 11^. The timing of this transition varies between organisms. Activation occurs as early as the 1- to 2-cell stage in mouse, between the 4- and 16-cell stages in most other mammals, and as late as cell divisions 6 to 8 in *Drosophila* and zebrafish ^9^. In addition to dramatic transcriptional changes, MZT coincides with other important changes, including lengthening of the cell cycle, emergence of cell cycle check points, and the capacity for cells to undergo apoptosis ^9, 11^.

The transition from a fully quiescent to active genome during MZT offers a unique and exciting opportunity to tease apart the relationship between chromatin and transcription. In zebrafish, the bulk of zygotic transcription starts after the 10th cell division at the 1000-cell stage (3 hours post fertilization [hpf]) ^4^. However, a minor wave of zygotic genome activation (ZGA) precedes this stage, and the earliest zebrafish transcripts emerge from the miR-430 microRNA gene cluster at the 64-cell stage (2 hpf) ^11– 17^. MicroRNAs from this cluster in turn play a key role in the degradation of maternally loaded transcripts during MZT ^18^.

### Core transcription factors drive zygotic genome activation

Initiation of zygotic transcription is generally mediated by a small number of pioneer factors, although the specific factors involved differ between species ^19– 24^. In zebrafish, transcription factors Pou5f3, Nanog, and SoxB1 proteins are critical for ZGA, binding to thousands of putative regulatory elements during this period ^14, 25– 27^. These transcription factors appear to be involved in nucleosome displacement through a two-step process. Before ZGA, they provide non-specific competition with histones on strong nucleosome footprints and then at ZGA they act synergistically to maintain open chromatin at regions with high nucleosome affinity ^28, 29^. Binding is associated with increased accessibility at MZT ^29, 30^, and recent work suggests that accessibility precedes and is predictive of future transcription ^31^.

During egg production, many mRNAs and proteins required for early development are maternally deposited. The extent to which early transcription factors are maternally loaded at the protein level is currently unclear. However, mRNAs encoding key transcription factors, including Pou5f3, Nanog, and SoxB1 proteins, are detected in embryos prior to the 64-cell stage, indicating that they are maternally loaded ^14, 25, 27^. Injection of translation-blocking morpholinos for either Pou5f3 or SoxB1 prevents ZGA ^14^, suggesting that translational regulation of these RNAs likely contributes to the control of ZGA.

### Additional signals are required for zygotic genome activation

The exact sequence of events that leads to ZGA is not well understood and remains an area of intense investigation. In addition to translational regulation of transcription factors, depletion of repressors, accumulation of activators, and local changes in chromatin accessibility have been implicated in promoting ZGA ^9, 11^. Of particular note, recent studies in Xenopus suggest that the concentration of histones in the early embryo may be critical for ZGA ^32– 36^. This also appears to be the case in zebrafish, as injection of core histones into the early zebrafish embryo is sufficient to delay ZGA whereas histone depletion accelerates ZGA ^36^. The concentration of histones on DNA does not change during the period leading up to ZGA, but the non-DNA-bound core histone concentration is decreased by early cleavage divisions ^36^. This results in a high nucleus-to-cytoplasmic ratio of histones at ZGA. This observation has led to a model in which reduced concentrations of unbound histones allow key transcription factors to successfully compete for DNA binding, thereby initiating ZGA ^29, 36, 37^. One challenge to this model is that zebrafish embryos injected with mRNA encoding the cell cycle regulator Chk1 arrest development between the 4- and 16-cell stages and maintain a low overall nuclear-to-cytoplasmic ratio yet these embryos are still able to activate a subset of zygotic genes ^38^. Although further investigation is needed, short genes seem to be less affected by the nuclear-to-cytoplasmic ratio during embryogenesis compared with long genes ^38, 39^. This raises the possibility that ZGA is differentially regulated at different subclasses of sequences.

Intriguingly, in zebrafish, early replication of “first wave” zygotic genes precedes initiation of their transcription. This finding raises the possibility that DNA replication is controlled by the same factors that poise these genes for transcription or that the pre-MZT replication timing program itself helps to prime early ZGA ^6^.

## The changing chromatin landscape during maternal-to-zygotic transition

**Figure 1.**
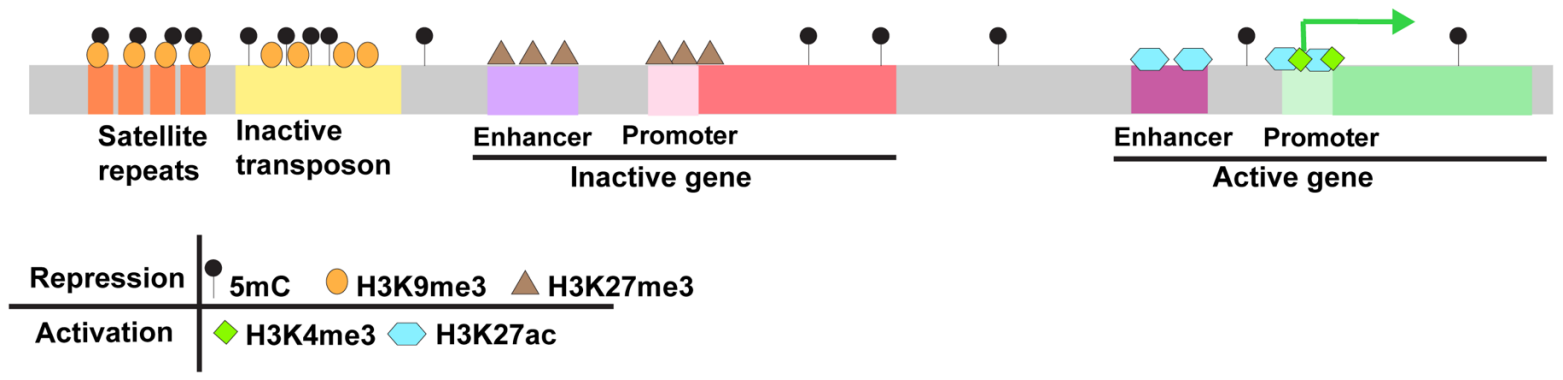
DNA methylation and histone modifications at transcriptionally active and repressed sequences.

**Figure 2.**
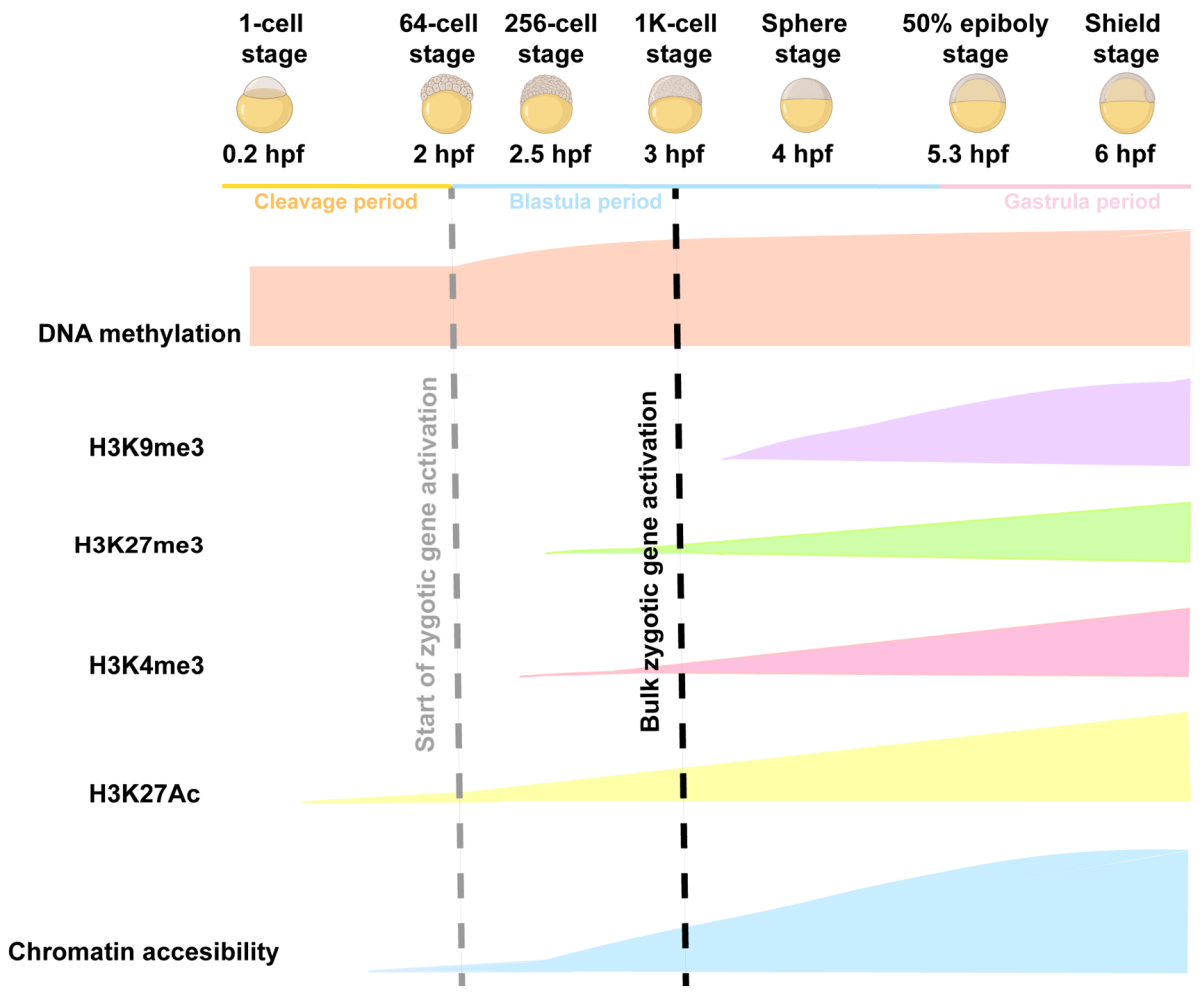
Dynamic changes in DNA methylation, histone modifications, and chromatin structure during early zebrafish development. hpf, hours post fertilization.

The relatively large number of cell divisions occurring between fertilization and ZGA has made zebrafish an appealing model for profiling the chromatin changes during this critical period. Genome-wide changes in DNA methylation, histone modifications, and chromatin structure have all been profiled at high temporal resolution during early zebrafish embryogenesis ( Figure 1 and Figure 2). As in other species, the early zebrafish genome generally exhibits features of open chromatin, which become increasingly constrained as development progresses. However, recent work has also uncovered intriguing differences between the early chromatin landscape in zebrafish and other models.

### 5-methylcytosine

5-methylcytosine (5mC) is the most common DNA modification in vertebrate genomes. It is associated with transcriptional repression and predominates at CpG dinucleotides ^40– 44^. Most CpGs in vertebrate genomes are methylated; the primary exception consists of non-methylated islands that generally overlap with promoters and other cis regulatory elements ^45– 48^. 5mC is essential for viability in vertebrates, and global loss of 5mC results in lethality in mice, frogs, and zebrafish ^49– 60^. Vertebrate 5mC is established by *de novo* DNA methyltransferases of the Dnmt3 family and maintained by the maintenance DNA methyltransferase Dnmt1 and its cofactor Uhrf1 ^61^. In addition to encoding Dnmt1 and Uhrf1, the zebrafish genome encodes six Dnmt3 orthologs, which exhibit differential expression during development ^62– 69^. Zebrafish also harbor orthologs of the 5mC dioxygenases Tet1, Tet2, and Tet3, which can promote active 5mC removal through the iterative oxidation of 5mC ^70– 73^.

The dynamics of DNA methylation are considerably different in zebrafish and mammals. The mammalian methylome is erased and re-established during preimplantation development and primordial germ cell formation ^42, 74– 83^. In contrast, zebrafish and other non-mammalian vertebrates do not appear to undergo similar large-scale demethylation ^46, 84– 91^. Instead, at least in zebrafish, the developing embryo adopts the 5mC landscape of the paternally inherited genome through gradual refashioning of the maternal methylome ^46, 88^. Surprisingly, the paternal genome is not required for this process ^46^. The specific *de novo* methyltransferases involved in establishing 5mC on the maternal methylome during this window have yet to be identified. Regions of methylation loss most likely undergo passive demethylation as Tet enzymes and oxidative derivatives of 5mC are undetectable during early zebrafish development ^46, 73, 88, 92– 95^.

The absence of Tets in zebrafish during early embryogenesis is in contrast to mammals, where Tets play a critical role in shaping the early embryonic methylome ^92^. As in mammals, Tet enzymes are important for demethylation of enhancer chromatin at later stages of zebrafish development ^94, 96, 97^. Very recent work suggests that 5mC patterns are also broadly stable in the zebrafish germline, although there is one curious exception ^90, 91^. There appears to be female-specific germline amplification and demethylation of an 11.5-kb repeat region encoding 45S ribosomal RNA ^91^. The failure to undergo large-scale erasure and re-establishment of 5mC in the zebrafish embryo or the germline may explain the transgenerational accumulation of 5mC at transgenes in zebrafish ^98, 99^. These observations also raise the possibility that inherited 5mC drives epiallelic regulation of endogenous zebrafish genes in some contexts.

### Histone modifications

Modification of the N-terminal tails of histones plays an instrumental role in shaping genomes into regions that are restrictive or permissive for transcription ^100– 102^. Unlike mammalian sperm, where the bulk of histones are replaced with protamine, zebrafish sperm rely entirely on histones to package their DNA ^103, 104^. In contrast to 5mC, most histone modifications undergo erasure and re-establishment during early zebrafish development, although reprogrammed histone modifications do not necessarily match the pattern of either gamete ^27, 28, 105– 109^.

***H3K27ac.*** One of the earliest histone modifications detected in the developing zebrafish embryo is histone H3 lysine 27 acetylation (H3K27ac), a modification associated with transcriptional activation ^27^. Deposition of H3K27ac is associated with initial access to promoters for early transcribed genes, and the highest H3K27ac enrichment occurs at Pou5f3-, SoxB1-, and Nanog-primed loci at ZGA ^27, 31^. Maternal depletion of the histone acetyltransferases *ep300b*, *crebbpa*, and *crebbpb* reduces detectable zygotic transcripts at the dome stage (4.3 hpf). Conversely, premature increases in the histone acetyltransferases Brd4 and P300 are sufficient to prematurely activate zygotic transcription ^38^. Together, these findings suggest a fundamental requirement for H3K27ac in promoting transcription during ZGA. Mechanistically, it is likely that increased histone acetylation during MZT helps relieve the repressive activity of histones, thereby promoting zygotic transcription ^38^.

***H3K4me3/H3K27me3.*** Trimethylation of histone H3 lysine 4 (H3K4me3) and trimethylation of histone H3 lysine 27 (H3K27me3) have important antagonistic roles in transcriptional regulation. H3K4me3 is typically associated with activation whereas H3K27me3 is associated with repression. A third class of bivalently marked genes harbor both modifications and exist in a state that is poised for transcription ^110– 113^. In zebrafish, H3K4me3 and H3K27me3 are present in sperm but are erased in the early embryo ^27, 104, 106, 107, 109^. Early chromatin microarray experiments revealed colocalized enrichment of H3K4me3 and H3K27me3 at promoters and transcriptional start sites at ZGA and these findings have recently been confirmed using chromatin immunoprecipitation followed by deep sequencing (ChIP-seq) ^27, 106, 107, 109^. Only a subset of genes marked by H3K4me3 or H3K4me3/H3K27me3 are actively transcribed at ZGA, and many bivalently marked genes remain poised for later expression. In addition, a large class of promoters is marked only by H3K4me3 in sperm and the pre-ZGA embryo. Some of these become bivalent after ZGA. Other genes are newly marked exclusively with H3K4me3 at ZGA ^106, 107, 109^. Large-scale resetting of H3K4me3 and H3K27me3 is also observed in early mammalian embryos ^114– 117^.

Somewhat surprisingly, H3K27me3-mediated repression may have only limited roles in the zebrafish embryo. Mouse mutants for the H3K27 methyltransferase Ezh2 die during embryogenesis between 7.5 and 10.5 days post coitum ^118^. However, although maternal/zygotic zebrafish mutants lacking this enzyme exhibit global depletion of H3K27me3, they show only limited changes in early gene expression, develop normally through gastrulation, and form a normal body plan ^119– 121^.

***H3K9me3.*** Trimethylation of histone H3 lysine 9 (H3K9me3) is enriched at repetitive sequences, including transposons, pericentromeric satellite sequences, telomeres, and some gene clusters ^122^. H3K9me3 is an essential component of highly condensed constitutive heterochromatin, which silences expression from repetitive sequences ^122^. H3K9me3 is broadly depleted in early mouse, worm, fly, and zebrafish embryos, and large-scale establishment follows ZGA ^108, 123– 125^. In zebrafish, the establishment of H3K9me3 appears to rely on miR430-mediated degradation of maternally loaded Smarca2, an ATP-dependent chromatin remodeling protein typically associated with the BAF complex ^108^. Other mechanisms have been proposed in flies and worms ^123, 126^. The lack of H3K9me3-marked heterochromatin in early embryos is somewhat surprising, as H3K9me3 is thought to promote genome stability ^122, 127^. The mechanisms that allow early embryos to maintain genome integrity in the absence of H3K9me3-marked constitutive heterochromatin are not known.

### Structural changes

Beyond the direct modification of histones and DNA, profound structural changes are observed as the embryonic genome passes through early development ^128, 129^. Chromatin structure can be visualized on multiple levels that reflect distinct aspects of DNA packaging. Structure in the zebrafish embryo has been recently assessed at the level of local chromatin accessibility by assay for transposase-accessible chromatin using sequencing (ATAC-seq), at the level of chromatin interactions by high-throughput chromatin conformation capture (Hi-C), and at the level of cellular ultrastructure by transmission electron microscopy (TEM).

***Accessibility.*** Fine-scale structural organization of chromatin is achieved through nucleosome positioning, which can promote or constrain transcription factor access to DNA. In zebrafish, prior to the major wave of ZGA, nucleosomes already appear positioned downstream of zygotic transcriptional start sites. During ZGA, these nucleosomes become organized into regular, well-positioned arrays near gene promoters ^28, 130^. Accessibility, as assessed by ATAC-seq, is detected at a small subset of sequences, including the miR-430 cluster at the 64-cell stage, but far more regions of accessibility emerge by the 1000-cell stage. At the onset of the major wave of ZGA, the majority of accessible regions are in promoters whereas by the dome stage accessibility at distal regulatory regions predominates ^30, 31^.

***Compartments and topologically associated domains.*** Hi-C–based mapping of chromatin interactions in somatic cells has revealed two levels of organization. First, chromatin can be segregated into distinct compartments; the A compartments contain transcriptionally permissive chromatin, and B compartments contain repressed chromatin ^131^. Compartments can be further subdivided into genomic regions known as topologically associated domains (TADs), which are thought to serve as regulatory scaffolds ^131– 133^. At 24 hpf, zebrafish have A/B compartments and TADs with genomic features similar to those observed in mammals, including enrichment of binding sites for the regulatory factor CTCF at TAD borders ^134– 138^. In most studied organisms, there is an absence of both compartments and TADs in early embryogenesis and these structures emerge during the major wave of ZGA ^135, 136, 139, 140^. In contrast, Hi-C in zebrafish reveals evidence of compartments and TADs at the 128-cell stage (2.25 hpf) prior to the major wave of ZGA. These structural features are lost during major ZGA and then re-established as embryonic development progresses ^134^. The mechanisms that drive these very early chromatin interactions are not clear, nor are the reasons that TADs dissolve at ZGA. There is also a possibility that formation of karyomeres in the early embryo impacts TAD organization, although this has not been explored experimentally. Along with CTCF, the cohesin complex has been implicated in TAD formation. Intriguingly, there is global shift in the genomic distribution of the cohesion complex component Rad21 during the window in which TADs are remodeled in zebrafish, raising the possibility that there is a causative relationship between these events ^141– 143^.

***Ultrastructure.*** TEM provides an additional approach for visualization of condensed chromatin ultrastructure. By this approach, condensed chromatin regions appear as electron-dense aggregates within cell nuclei. Consistent with the lack of histone modifications associated with heterochromatin, early zebrafish embryos also lack condensed chromatin ultrastructure. Just prior to the major wave of ZGA, at the 512-cell stage, embryonic nuclei are completely devoid of the electron-dense aggregates, and aggregates similar to those classically observed in somatic cells emerge in all embryonic nuclei between 3.7 and 6 hpf ^108^. This time line of compaction correlates with H3K9me3 establishment in the embryo ^108^. The clear presence of A and B compartments in zebrafish embryos by Hi-C at the 128-cell stage is curious in light of the lack of ultrastructure visualized by TEM at the 512-cell stage. Future analysis will be required to determine whether differences reflect minor discrepancies in timing between analyses or rather suggest that early Hi-C interactions are insufficient to drive chromatin segregation at the ultrastructure level.

## Conceptual advances and emerging directions

### Conceptual advances

In addition to foundational work profiling chromatin changes during embryogenesis, a number of important conceptual advances have emerged from recent studies in zebrafish. Among these is the concept of the placeholder nucleosomes, which enable programming of DNA methylation on the maternal genome during early embryogenesis and prime promoters for later expression. Placeholder nucleosomes contain the histone H2A variant H2A.Z and H3 histones that are monomethylated on lysine 4 (H3K4me1). In both sperm and cleavage-stage zebrafish embryos, placeholder nucleosomes occupy virtually all regions lacking DNA methylation, and perturbation of these placeholders causes expansion of 5mC domains ^105, 144^. At ZGA, genes marked by placeholders become either active and marked by H3K4me3 and H3K27ac or silenced and marked by H3K4me3/H3K27me3. The accumulation of these modifications suggests that these specialized nucleosomes poise gene promoters in an unmethylated state that can be readily activated at ZGA or during later development ^27, 105^. The presence of placeholders also suggests a clear mechanism by which methylation of the maternal methylome is remodeled during embryogenesis in the absence of information from the paternal methylome ^105^.

Another emerging theme arising from work in zebrafish is the importance of maternally loaded RNA and protein in shaping the early chromatin landscape. Depletion of maternally loaded histones is implicated in activation of zygotic transcription, while clearance of the maternal factor Smarca2 is required for H3K9me3 establishment and condensed chromatin ultrastructure ^36, 108^. Recent evidence also suggests that resolution of primed promoters into active or silenced states relies predominately on maternal factors, although the specific factors involved have not been identified ^27^. Around one third of zebrafish embryonic transcripts are exclusively maternal, whereas most additional transcripts are expressed both maternally and zygotically ^16^. These RNA pools include transcripts for many candidate chromatin regulators. To date, one challenge to understanding the function of maternally loaded gene products has been the need to generate maternal/zygotic mutant embryos. For genes that are also required later in development, generation of maternal zygotic mutants often requires the labor-intensive process of germ cell transplantation, which can present an obstacle to rapid progress ^145^. New technologies for germline-specific CRIPSR/CAS9-mediated mutation or degradation of maternally loaded gene products in the embryo are expected to aid in probing the functions of these maternally loaded factors ^146– 151^.

### Emerging strategies

The ability to readily manipulate the chromosomal content of the early embryo provides a unique tool for probing early chromatin regulation. Haploid zebrafish embryos are relatively normal during the first day of development, and triploid zebrafish are viable to adulthood ^152– 155^. Haploid embryos have been elegantly used to demonstrate that the female methylome can reset without any input from male chromosomes and to show that decreased DNA content leads to delayed ZGA ^38, 46^. The recent discovery of the Ly6/uPAR protein Bouncer as necessary for species-specific fertilization in fish also opens up the opportunity to make hybrid embryos from medaka sperm and zebrafish eggs ^156^. A detailed assessment of ZGA or the early chromatin environment has yet to be undertaken in these embryos, but they offer a unique system in which to probe DNA intrinsic versus extrinsic factors that contribute to the shifting landscape during early embryogenesis.

In addition to the ability to manipulate chromosome content, ** the clarity and accessibility of the zebrafish embryo provide an exciting opportunity to visualize zygotic transcription and associated chromatin dynamics in intact embryos. Visualization of newly synthesized transcripts in the early embryo can be achieved by 5-ethynyl uridine (EU) labeling bulk RNA followed by click chemistry or through targeted visualization of highly expressed RNAs using fluorophore-conjugated morpholinos ^17, 38^. At the same time, CRISPR-dCAS9-GFP complexes can be used to visualize specific DNA loci within embryonic nuclei ^38^. With these approaches, two foci of miR-430 expression, corresponding to the two chromosomal miR-430 gene clusters, can be visualized in a small fraction of embryonic nuclei beginning at the 64-cell stage, and all nuclei show two miR-430 foci by the 512-cell stage ^17, 38^. In addition to containing miR-430 transcripts, foci contain activated RNA polymerase II and RNA transcripts derived from gene clusters encoding zinc finger transcription factors ^17^. Signal from these transcriptional hubs appears to be specific to the early embryo as they dissolve after the major wave of ZGA ^6^.

Injection of fluorescently labeled modification-specific antigen-binding fragments (Fabs) provides an additional new tool, allowing monitoring of chromatin changes in live zebrafish embryos ^157– 159^. By this approach, H3K27ac is observed in two nuclear foci corresponding to the miR-430 gene clusters in 64- to 1000-cell stage embryos. Intriguingly, H3K27ac still appears at these foci when zygotic transcription is inhibited, suggesting that the establishment of H3K27ac at these foci precedes activation of transcription ^157^.

Additional new technologies for visualizing chromatin and transcription in the early zebrafish embryo continue to emerge. Light-sheet microscopy tracking transcription factors, including Sox19b, was recently used to demonstrate that the chromatin-bound fraction of transcription factors increases over early embryonic cell divisions. Furthermore, new preprinted work visualizing transcriptional activity by three-color stimulated emission depletion (STED) super-resolution and live-cell microscopy suggests that, after transcription initiates, regions of active euchromatin form RNA-enriched microenvironments that exclude inactive euchromatin ^37, 160^.

## Conclusions

The past few years have led to an explosion in articles exploiting the zebrafish system to understand chromatin dynamics and their relationship to transcription during the initial activation of the zygotic genome. Recent high-resolution profiling of DNA, histone modification, and structural changes occurring during this period offer a critical foundation for understanding how these many signals are integrated in the early embryo, while new technologies that are well suited to the zebrafish model offer the opportunity for continued advances.
